# ERK and AKT phosphorylation status in lung cancer and emphysema using nanocapillary isoelectric focusing

**DOI:** 10.1136/bmjresp-2015-000114

**Published:** 2016-02-17

**Authors:** Philip A J Crosbie, Emma J Crosbie, Mark Aspinall-O'Dea, Michael Walker, Rebecca Harrison, Maria Pernemalm, Rajesh Shah, Leena Joseph, Richard Booton, Andrew Pierce, Anthony D Whetton

**Affiliations:** 1 North West Lung Centre, University Hospital of South Manchester, Manchester, UK; 2 Stem Cell and Leukaemia Proteomics Laboratory, University of Manchester, Manchester Academic Health Science Centre, Manchester, UK; 3 Institute of Cancer Sciences, University of Manchester, St Mary's Hospital, Manchester, UK; 4 Manchester Medical School, University of Manchester, Manchester, UK; 5 Department of Oncology and Pathology, Karolinska Institutet, SciLifeLab, Stockholm, Sweden; 6 Department of Thoracic Surgery, University Hospital of South Manchester, Manchester, UK; 7 Department of Pathology, University Hospital of South Manchester, Manchester, UK

**Keywords:** Lung Cancer, Emphysema, Non-Small Cell Lung Cancer

## Abstract

**Background:**

Emphysema is an independent risk factor for the development of lung cancer in smokers. Activation of oncogenic signalling proteins AKT and ERK by phosphorylation has an established role in the development of lung cancer and has also been implicated in the pathogenesis of emphysema. The aim of this study was to compare the protein level and phosphorylation status of AKT and ERK in paired lung cancer and emphysema tissue using a highly sensitive phosphoprotein analysis approach.

**Methods:**

An antibody-based, nanocapillary isoelectric focusing (cIEF) assay was used to determine the relative quantities and phosphorylation status of AKT and ERK in tumour and matched lung tissue from patients, with or without evidence of emphysema, undergoing curative resection for non-small cell lung cancer.

**Results:**

20 patients with adenocarcinoma (n=9) or squamous cell carcinoma (n=11) of the lung were included (mean age 67.3 years (SD 7.5, range 47–80 years)), 12 were men and all were current (n=10) or former smokers (n=10). Paired macroscopically normal lung tissue was either histologically normal (n=7) or showed emphysema (n=13). Total and phosphorylated AKT levels were fourfold (p=0.0001) and fivefold (p=0.001) higher in tumour compared with matched lung, respectively. There was no correlation with tumour histology, stage or differentiation; however, total AKT signal in tumour was significantly correlated with fluorodeoxyglucose avidity on positron emission tomography-CT scan (r=0.53, p=0.035). Total ERK was not differentially expressed, but doubly phosphorylated (activated) ERK was threefold higher in emphysema (23.5%, SD 9.2) than either matched tumour (8.8%, SD 8.6) or normal lung tissue (8.3%, SD 9.0) and correlated with the histological severity of emphysema (p=0.005).

**Conclusions:**

cIEF offers opportunities for quantifying subtle shifts in the phosphorylation status of oncoproteins in nanogram amounts of lung tissue. ERK activation is a feature of emphysema.

**Key messages**

- ERK activation, through double phosphorylation, is a feature of emphysema.
- Capillary isoelectric focusing offers opportunities for quantifying subtle shifts in the phosphorylation status of oncoproteins in nanogram amounts of lung tissue.
- Total and phosphorylated AKT is over expressed in tumour than matched normal lung.

## Introduction

Lung cancer is the leading cause of cancer-related death in the world, responsible for 1.6 million deaths/year.^1^ The major risk factor for the development of lung cancer is chronic exposure to tobacco smoke.^2^ This risk is significantly increased in smokers who have co-existent chronic obstructive pulmonary disease (COPD);^3–5^ lung cancer is a leading cause of death in this population.^6–9^ COPD, which encompasses a heterogeneous group of disorders that include chronic bronchitis and emphysema, is associated with chronic inflammation^10^ and it is postulated that inflammation is an important driver of lung carcinogenesis.^11^ Exploring the common molecular pathways between these smoking-related conditions may provide insights into mechanisms of disease and so help to improve outcomes for both.

Dysregulation of the AKT and ERK signalling cascades has been implicated in malignant transformation.^12–14^ Sustained activation by phosphorylation results in aberrant signalling that facilitates not only cellular proliferation, but drives tumour invasion^15^ and prolongs cancer cell survival.^16^ Previous non-small cell lung cancer (NSCLC) studies have reported the presence of phosphorylated AKT in 33–79% of tumours^17–22^ and identified it as a key determinant of tumour aggressiveness associated with poor survival.^19^ ^21^ ^23^ ERK isoforms (1 and 2) are key modulators of cell proliferation.^24^ Phosphorylation of both threonine (Thr202) and tyrosine (Tyr204) residues (double phosphorylation) are required for full kinase activity; removal of one phospho group (monophosphorylation) or both inactivates the enzyme.^24^ Activating K-RAS mutations promote constitutive ERK phosphorylation, leading to uncontrolled cellular proliferation. K-RAS mutations have been detected in approximately 20% of NSCLC, particularly adenocarcinomas.^25^ Phosphorylated ERK has been detected in up to one-third of NSCLCs with an inconsistent association with prognosis.^22^ ^26^ ^27^ Elevated phosphorylated ERK has also been reported in emphysema compared with healthy lung tissue,^28^ and it is postulated that constitutive ERK activation may be a critical event in emphysema progression.^29^ ^30^ Evidence also points to activation of the PI3K/AKT/mTOR pathway in the pathogenesis of COPD.^31^ ^32^

Nanocapillary isoelectric focusing (cIEF), first reported by O'Neill *et al*^33^ to detect low levels of signalling proteins in just 25 cells, was used in this study to determine the relative protein level and phosphorylation status of ERK and AKT in lysates from tumour and matched lung tissue in patients with and without pathological evidence of emphysema. The precise resolution of proteins allows post-translational modifications like phosphorylation to be reproducibly detected in nanograms of total protein.^34^ ^35–38^

## Materials and methods

### Study participants

Patients scheduled to undergo curative-intent surgical resection for NSCLC were recruited from the Department of Thoracic Surgery at the University Hospital of South Manchester between February 2011 and November 2011. All patients completed a risk factor questionnaire prior to surgery detailing smoking exposure and medical history. Patients with a history of malignancy (within 5 years) were excluded from the study. Preoperative spirometry was recorded in all patients and included forced expiratory volume in 1 s (FEV_1_) and forced vital capacity (FVC). COPD was defined as FEV_1_/FVC <70% and severity classified according to the global initiative for chronic obstructive lung disease (GOLD).^39^ The study was approved by the local ethics committee (Reference 10/H1008/79) and all participants provided written informed consent.

### Lung sample collection

Lung specimens were sampled immediately after surgical resection by a specialist thoracic pathologist, with samples taken from the tumour and from adjacent macroscopically normal lung tissue. Biopsies were frozen within 1 h of lung resection and stored at −80°C. Thorough pathological examination of resected lung tissue was performed and findings recorded in accordance with the seventh edition of the Tumour Node Metastasis (TNM) Lung Cancer Staging classification.^40^ Macroscopically normal lung tissue was also examined and the presence and severity of emphysema recorded.

### Sample preparation

Frozen lung tissue was crushed in liquid nitrogen using a pestle and mortar resting on a bed of dry ice and then lysed at 4°C with Bicine/CHAPS lysis buffer (Protein Simple, Santa Clara, California, USA) for 4 h with regular mixing. Lysates were centrifuged at 18 407 g for 15 min at 4°C to remove debris and the supernatant preserved. The protein concentration of each cell lysate was determined using a Bradford protein assay (Biorad) and the samples were normalised to 2 mg/mL. The cell lysates were diluted to 0.4 mg/mL with sample diluent (Protein Simple, Santa Clara, California, USA), mixed in a 1:3 ratio with ampholyte (pH range of 5–8 nested) and fluorescent ladder premix (Protein Simple, Santa Clara, California, USA) and placed in quadruplicate wells of a 384-well microplate at 0.1 mg/mL.

### cIEF assay

The assay was performed as previously described using the NanoPro 1000 platform (Protein Simple, Santa Clara, California, USA).^34^ In brief, 40 ng total protein (whole tissue lysate) was loaded per capillary. Sample lysates underwent isoelectric focusing (40 min separation at 21 000 μW) followed by ultraviolet-mediated protein fixation (80 s). ERK1/2, phospho-ERK1/2, ERK1 (Protein Simple, Santa Clara, California, USA) and AKT (Cell Signalling Technology, Danvers, Massachusetts, USA) primary antibodies were used at a 1:50 dilution; the secondary (goat antirabbit biotin) and tertiary (streptavidin-HRP) antibodies were used at 1:100 dilution in antibody diluent (Protein Simple, Santa Clara, California, USA). Primary incubation times were 120 min for ERK and 240 min for AKT antibodies. Secondary and tertiary incubation times were 60 and 10 min, respectively, and the standard washing protocol was employed between steps. The detection of chemiluminescence involved a 240 s exposure. A pool of the samples normalised to 0.1 mg/mL was used as a positive control and also as quality assurance for assay-to-assay variability.

### Peak analysis

**Figure 1:**
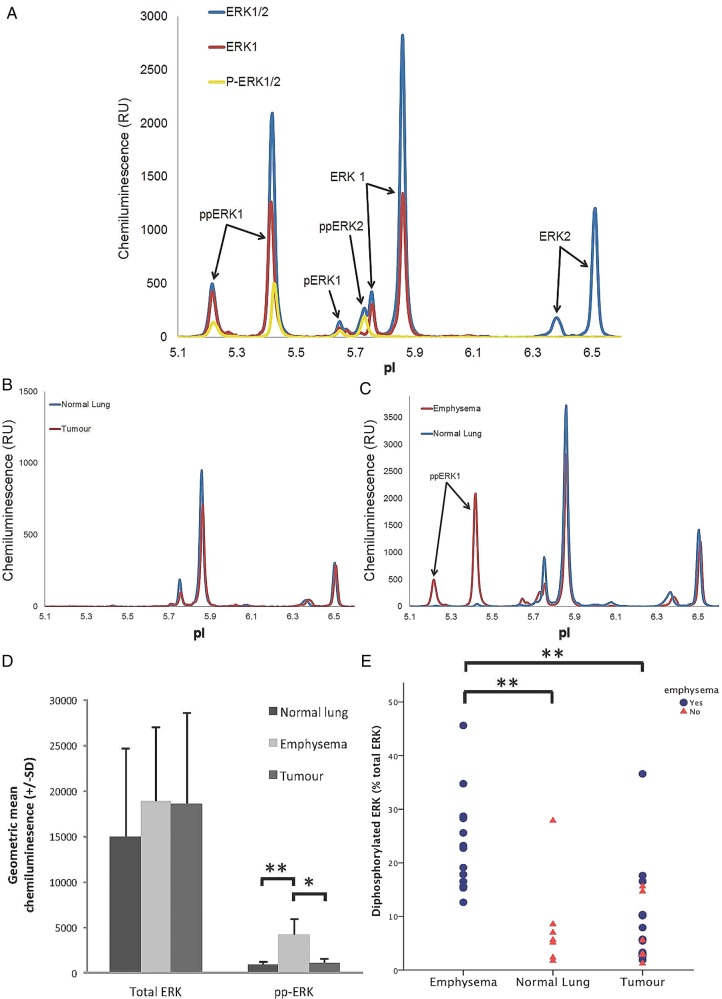
(A) ERK spectra peak identification using three different primary antibodies (ERK1/2, ERK1 and phospho-ERK1/2) in one sample (emphysema) allowing accurate detection of the various phosphorylated isoforms of ERK1/2 (pp=doubly phosphorylated (active isoform), p=monophosphorylated (inactive) and non-phosphorylated (inactive)). (B and C) Representative peaks using ERK1/2 primary antibody in matched normal lung and tumour, and emphysema and normal lung, respectively. (D) Bar chart showing geometric mean total ERK1/2 and total doubly phosphorylated ERK1/2 stratified by tissue type (normal lung, emphysema and tumour). (E) Proportion of ERK1/2 doubly phosphorylated according to tissue type (emphysema, normal lung and tumour). RU, relative unit; pI, isoelectric point.

Spectral peaks were numbered according to isoelectric point (pI); the pI is the pH at which individual protein residues remain stationary in the pH gradient when applying an electrical charge. Increasing phosphorylation results in decreasing pI values. Peaks at specific pIs corresponded to native ERK1 and 2 isoforms and phosphorylated equivalents including doubly phosphorylated ERK (ppERK), the fully active isoform, and monophosphorylated or non-phosphorylated ERK (pERK and ERK, respectively), which are inactive (figure 1A). Exposure of A549 cells to epidermal growth factor (EGF) in cell culture increased the ppERK proportion of total ERK from a baseline of 31.5–59.3% (see online supplementary figure S1). AKT produced a complex signal that included eight spectral elements across a narrow range of pI values. Analysis of AKT peaks was informed by the work of Iacovides *et al*^36^ and by analysis of the AKT peak spectrum of A549 cells cultured with and without EGF stimulation. EGF stimulation resulted in a shift of signal into the more acidic (lower pI) range, consistent with phosphorylation event(s) (see online supplementary figure S1). Phosphorylated AKT was therefore defined as the first four peaks of the AKT spectra.

10.1136/bmjresp-2015-000114.supp1 Supplementary figure 1

Peak area and percentage peak contribution was calculated for each peak using Compass software (V.1.8.0, Protein Simple). Peak area represented chemiluminescent signal expressed as relative units (RUs) allowing a relative comparison of protein level but not absolute quantification. There was a linear relationship between protein concentration and log chemiluminescence signal (see online supplementary figure S2). Total ERK and AKT protein in a sample was calculated by adding the area from all assigned peaks. Peak contribution to total area under all peaks was calculated and expressed as a percentage. ERK and AKT protein phosphostatus was assessed by comparing the ratio of putative phosphorylated to non-phosphorylated peaks with respect to area under the peaks and percentage peak contribution using Compass software (V.1.8.0, Protein Simple).

10.1136/bmjresp-2015-000114.supp2 Supplementary figure 2

### Assay performance

Assay performance was assessed using positive control samples (pooled lung and tumour lysate). The coefficient of variance (CV) was calculated for ERK (three main peaks) and AKT (six main peaks) assays in lung tissue across eight independent runs. CV for ERK peak analysis using percentage peak contribution was 3.3–9.0% and peak area 5.2–12.8%, for AKT percentage peak contribution CV was 2.9–15.8% and 9.4–25.1% for peak area. The limit of detection was determined using serial dilution of lung lysate; no peaks above background were seen at 0.01 mg/mL (4 ng total protein), discernable peaks were seen at 0.02 mg/mL, which was defined as the limit of detection (8 ng total protein) and reliable peak measurement using percentage peak contribution at 0.04 mg/mL (16 ng total protein), which defined the limit of quantitation.

### Statistical analyses

Data were analysed using SPSS statistics software package (V.20.0.0). Paired data (tumour and matched lung tissue) were analysed using the paired t test and unpaired data by the Student t test. Pearson's correlation coefficient was used to investigate the effects of tumour size on phosphorylation status of ERK1/2. A p value <0.05 was taken as statistically significant.

## Results

Twenty participants undergoing curative-intent resection of NSCLC were recruited into the study. Mean age 67.3 years (SD 7.5, range 47–80 years), 12 males and 8 females. All were current (n=10) or former smokers (n=10) with a mean smoking exposure of 48.4 pack years (SD 24.4) and a mean smoking duration of 42.5 years (SD 12.8). Mean FEV_1_ was 72.2% predicted (SD 21.0) and mean FEV_1_/FVC ratio 60.6% (SD 14.8). COPD was present in 14 patients and severity was classified as mild (GOLD stage 1, n=4), moderate (GOLD stage 2, n=8) or severe (GOLD stage 3, n=2). Tumour was located in the upper lobe of the lung in 12 participants and the lower lobe in 8. The mean tumour size was 43.2 mm (SD 20.4, range 19–90 mm). The final pathology was TNM stage I (n=9), stage II (n=9) and stage III (n=2) adenocarcinoma (n=9) or squamous cell carcinoma (n=11). Paired macroscopically normal lung tissue was either histologically normal (n=7) or showed emphysema (n=13), graded as mild (n=7), moderate (n=3) and severe (n=3). Pathological evidence of emphysema was present in 10/14 patients with COPD and in 3/6 patients without COPD. Histological severity of emphysema was correlated with airflow obstruction (FEV_1_/FVC: 66.4% (SD 14.9) in normal lung, 65.8% (SD 14.2) in mild, 55.0% (SD 1.7) in moderate and 42.0% (SD 5.3) in severe emphysema; one-way analysis of variance (ANOVA) p=0.055).

### ERK1/2 analysis

An ERK spectrum was detected in 19 out of 20 tumour samples and all 20 matched lung samples. A representative spectrum with peaks identified using ERK1/2, ERK1 and phospho-ERK1/2 primary antibodies is shown in figure 1A. One tumour sample was excluded from further analysis because of lack of signal for both ERK and AKT, possibly related to poor tissue quality. Total ERK did not differ significantly between tumour and matched lung (p=0.75, n=19; table 1). However, lung tissue had a significantly higher level of total doubly phosphorylated ERK1/2 than tumour when expressed either as total peak area (2630RU (SD 912) vs 1170RU (SD 355); p=0.006) or percentage peak contribution to total ERK (19.1% (SD 11.2; range 1.7–45.6%) vs 8.8% (SD 6.3; range 1.2–36.6%); p=0.002). Age, sex and smoking exposure, defined by smoking status, pack years or smoking duration, were not correlated with any measure of ERK in lung tissue. There was no correlation of ERK status with tumour site, histology or stage.

**Table 1:** Analysis of relative protein level and phosphorylation status of ERK 1/2 and AKT in lung and tumour tissue

	Lung vs matched tumour (±SD)			Squamous cell carcinoma vs adenocarinoma (±SD)			Emphysema vs histologically normal lung (±SD)		
	Lung	Tumour		Squamous	Adenocarcinoma		Emphysema	Normal	
Measured kinase	n=19	n=19	p Value*	n=10	n=9	p Value†	n=13	n=7	p Value†
Total ERK (RU)	17 179±8222	18 578±9886	0.75	19 815±10 399	17 298±9099	0.65	18 836±8147	14 997±9638	0.51
Total ppERK (RU)	2630±912	1170±355	0.006	1236±506	977±239	0.67	4159±1722	853±422	0.001
ppERK as per cent total ERK	19.1±11.2	8.8±8.6	0.002	8.3±6.3	9.3±11.1	0.80	23.5±9.2	8.3±9.0	0.002
Total AKT (RU)	6457±2673	25 645±10 447	0.0001	27 040±11 803	24 155±8750	0.79	7079±2748	6026±2780	0.70
Phospho-AKT (RU)	1932±511	10 186±3327	0.001	9204±3020	11 402 ±3516	0.69	2223±557	1706±490	0.68
pAKT as per cent total AKT	33.2±13.5	48.6±13.0	0.001	48.7±15.2	48.4±11.0	0.96	34.3±12.7	31.7±14.9	0.69

*Paired t test.

†Student t test.

pp, doubly phosphorylated.

No difference in any measure of ERK was seen in lung tissue according to COPD status or when classified according to severity (GOLD classification). Lung samples were then stratified according to histology into normal (n=7) and emphysematous lung for further analysis (n=13). There was no difference in total ERK1/2 between normal and emphysematous lung (18 836 RU (SD 8147) vs 14 997 RU (SD 9638), p=0.51; table 1). However, both the total amount of doubly phosphorylated ERK 1/2 (4159 RU (SD 1722) vs 853 RU (SD 422), p=0.001; a fivefold difference) and the proportion of total ERK1/2 that was doubly phosphorylated (23.5% (SD 9.2) vs 8.3% (SD 9.0), p=0.002; a threefold difference) was significantly higher in patients with histological evidence of emphysema compared with those with normal lung (table 1, figure 1B–E). Furthermore, the total amount (one-way ANOVA, p=0.002) and the proportion of ERK1/2 that was doubly phosphorylated (one-way ANOVA, p=0.005) increased with increasing severity of emphysema as assessed by histological criteria, suggesting a dose–response relationship.

### AKT analysis

**Figure 2:**
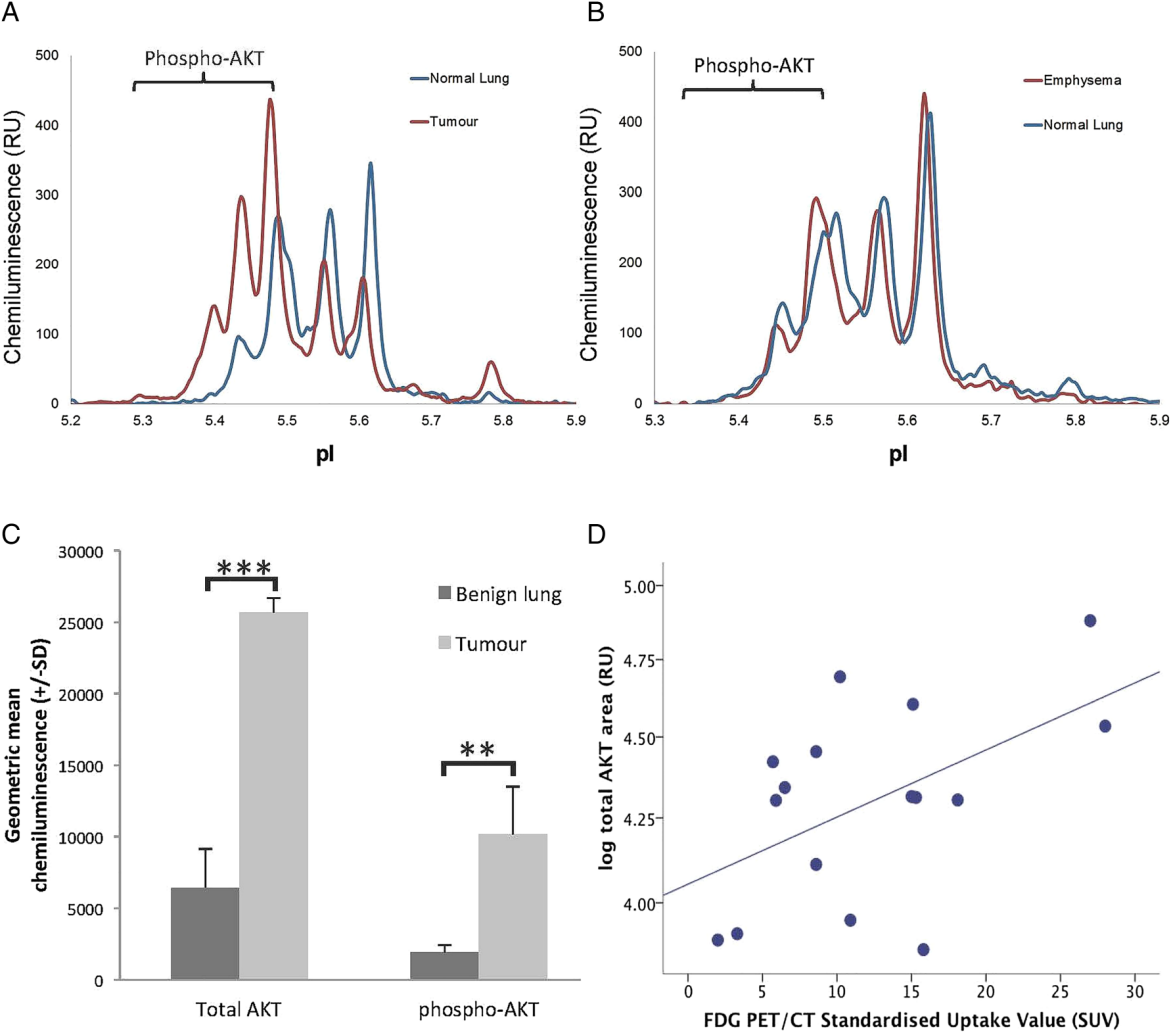
(A and B) Representative peaks using AKT primary antibody in normal lung and emphysema and tumour and matched normal lung, respectively. (C) Bar chart showing geometric mean total AKT and total phosphorylated AKT in benign lung and tumour tissue. (D) Correlation of total AKT signal with FDG PET/CT SUV. *p<0.05, **p<0.01, ***p<0.001. FDG, fluorodeoxyglucose; PET, positron emission tomography; RU, relative unit; SUV, standardised uptake volume.

An AKT spectrum was detected in 19 out of 20 tumour samples and all 20 matched lung samples. The relative level of both total AKT and phosphorylated AKT was markedly different between tumour and matched lung. Total AKT (25 645 RU (SD 10 447) vs 6457 RU (SD 2673), p=0.0001) and total phospho-AKT (10 186 RU (SD3327) vs 1932 RU (SD 511), p=0.001) were fourfold and fivefold higher in tumour, respectively (figure 2A–C). The proportion of AKT that was phosphorylated was also significantly higher in tumour (48.6% (SD 13.0) vs 33.2% (SD 13.5), p=0.001). AKT did not differ according to histological diagnosis or presence of emphysema (table 1).

AKT did not differ according to age or sex in either lung or tumour tissue. Total AKT was not related to smoking status. However, the proportion of phosphorylated AKT was higher in lung from current compared with former smokers (38.6% (SD 11.1) vs 28.1% (SD 13.5), p=0.074), a difference that approached significance. AKT was not related to smoking duration, pack years or alcohol intake. No difference in any measure of AKT was seen in lung tissue according to COPD status or the presence of emphysema. AKT status was also unrelated to tumour size, site, stage or degree of differentiation. However, total AKT was significantly correlated to tumour standardised uptake value (SUV) by fluorodeoxyglucose (FDG) positron emission tomography-CT (PET-CT) scanning (r=0.53, p=0.035; n=16; figure 2D); total phospho-AKT (p=0.76) and proportion of AKT phosphorylated (p=0.15) were not associated with tumour SUV.

## Discussion

### Summary of main findings

Total and phosphorylated AKT were fourfold and fivefold higher in tumour than matched normal lung, respectively. There was no correlation with tumour histology, stage or differentiation; however, total AKT signal was significantly correlated with FDG avidity on PET-CT scan, an independent prognostic factor in NSCLC.^41^ AKT phosphorylation was increased in lung tissue in current compared with former smokers, a difference that approached significance. The proportion of ERK that was doubly phosphorylated (active) was not increased in tumour but was threefold higher in emphysema than normal lung, and correlated with the histological severity of emphysema.

### Strengths and limitations

Here we describe a highly sensitive cIEF immunoassay used to define the phosphorylation status of ERK and AKT in NSCLC and matched lung. cIEF offers limits of detection and quantitation in the nanogram range of total protein from lung and tumour tissue. This sensitivity enables a detailed characterisation of phosphorylation status, including relative quantification and isoform delineation. There is considerable scope for this technology to be applied to clinical settings where biological sampling may be limited, for example, at bronchoscopy.

We sampled freshly resected lung tissue that was frozen immediately to minimise the chance of tissue autolysis and protein degradation.^42^ Paired macroscopically normal lung was sampled outwith the immediate vicinity of the tumour to avoid inadvertently sampling microscopic malignant cells. Any lung tissue adjacent to tumour cannot be regarded as entirely normal, as it is exposed to identical carcinogenic and genetic risk factors as the tumour itself, but detailed histopathological analysis excluded any microscopic neoplastic change. There has been much interest recently in the supporting role of the tissue microenvironment in tumorigenesis and it is possible that even microscopically normal cells have upregulated kinase activity by virtue of their location. Taking normal lung biopsies from healthy controls would avoid this potential source of bias, but these samples are difficult to acquire. The NSCLC sample was taken from the centre of the tumour to prevent contamination with normal lung cells. Even still, it is likely that the tumour sample included stromal tissues, blood and other cells that were not malignant, so the biopsies should be regarded as ‘tumour enriched’ rather than tumour per se. The specific isolation of tumour cells through laser capture microdissection would more accurately restrict analysis to our cells of interest and this may be considered for future studies.

### Findings in the context of previous studies

In the present study, there was a significant correlation between the phosphorylation status of ERK and the presence and histological severity of emphysema. Our results refine the observation presented by Mercer *et al*^28^ who demonstrated a twofold increase in phospho-ERK activity in emphysematous compared with healthy lungs, using western blot analysis. The authors hypothesised that a switch from transitory to constitutive ERK activation may be a critical event in emphysema progression by increasing the production of matrix metalloproteinase 1, which has been causally linked to the development to emphysema.^29^ ^30^ Furthermore, while acknowledging the importance of cigarette smoke exposure in the aetiology of emphysema, the authors were able to demonstrate the increased activity of ERK in emphysema to be independent of ongoing cigarette smoke inhalation; we also failed to find an association between phosphorylated ERK expression and smoking.

Our report of AKT overexpression and phosphorylation in tumour is supported by numerous previous studies in NSCLC, with high AKT and phospho-AKT expression generally associated with poor prognosis.^17^ ^21^ ^43^ AKT is also known to be a central player in cell metabolism and drives glycolytic activity in human tumours.^44^ We detected a correlation between AKT expression and FDG-PET signal in the tumours but not emphysema. Tumour FDG avidity has been reported to be an independent prognostic factor in patients with resectable NSCLC.^41^ ^45^

The presence of emphysema or COPD, of which emphysema forms a major subcategory, is by far the greatest risk factor for lung cancer in smokers.^3–5^ By analysing matched tissue from patients with lung cancer and emphysema, we have shown that two important oncogenic signalling pathways are differentially expressed. By analysing both diseases in the same patient, it is possible to control for environmental factors that might confound findings when analysing samples from different patients such as diet or smoking.

### Conclusions and future directions

This novel method offers an unparalleled opportunity to explore differences in the phosphorylation patterns of a variety of important proteins in tiny biological samples. We have shown that ERK and AKT have different phosphorylation profiles in tumour and emphysema. This insight requires further investigation. Defining the phosphostatus of key oncoproteins may identify novel targets for the treatment of lung cancer and emphysema, two of the most important causes of morbidity and mortality worldwide.
