# Supplementary material for: ERK and AKT phosphorylation status in lung cancer and emphysema using nanocapillary isoelectric focusing
Source: BMJ Open Respir Res. 2016 Feb 17;3(1):e000114. doi: 10.1136/bmjresp-2015-000114 (PMC4762086; doi:10.1136/bmjresp-2015-000114)
Supplement: Supplementary figure 1 [file supplement-figure1.pdf]

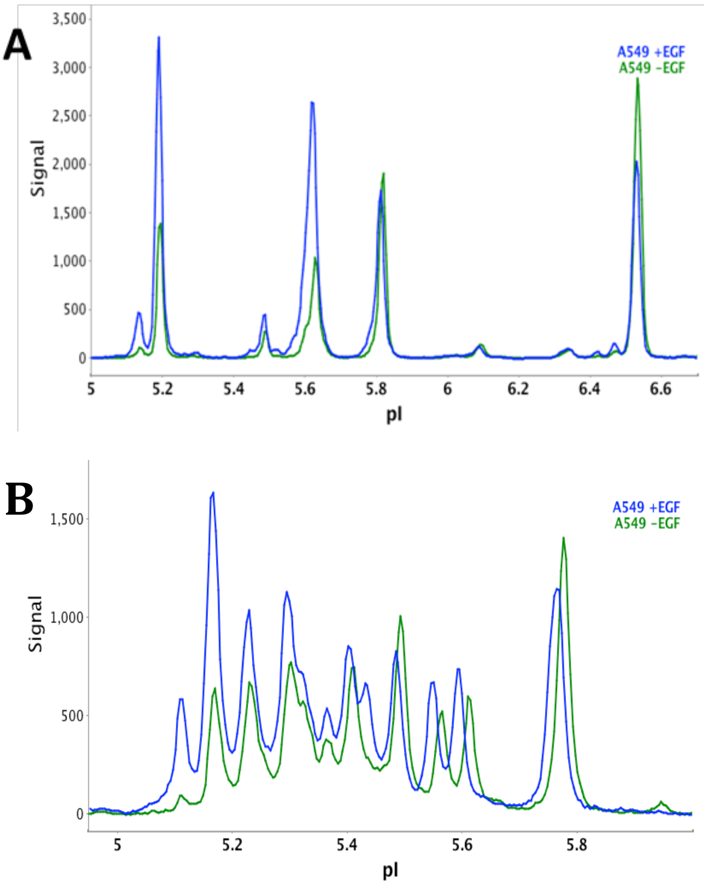

Supplementary Figure 1. Comparison of exposed (EGF+ve = blue) and unexposed (EGF-ve = green) A549 cells using **(A)** ERK1/2 and **(B)** AKT primary antibodies. Baseline pp-ERK proportion increased from 31.5% to 59.3% after EGF exposure.
