# Supplementary material for: ERK and AKT phosphorylation status in lung cancer and emphysema using nanocapillary isoelectric focusing
Source: BMJ Open Respir Res. 2016 Feb 17;3(1):e000114. doi: 10.1136/bmjresp-2015-000114 (PMC4762086; doi:10.1136/bmjresp-2015-000114)
Supplement: Supplementary figure 2 [file supplement-figure2.pdf]

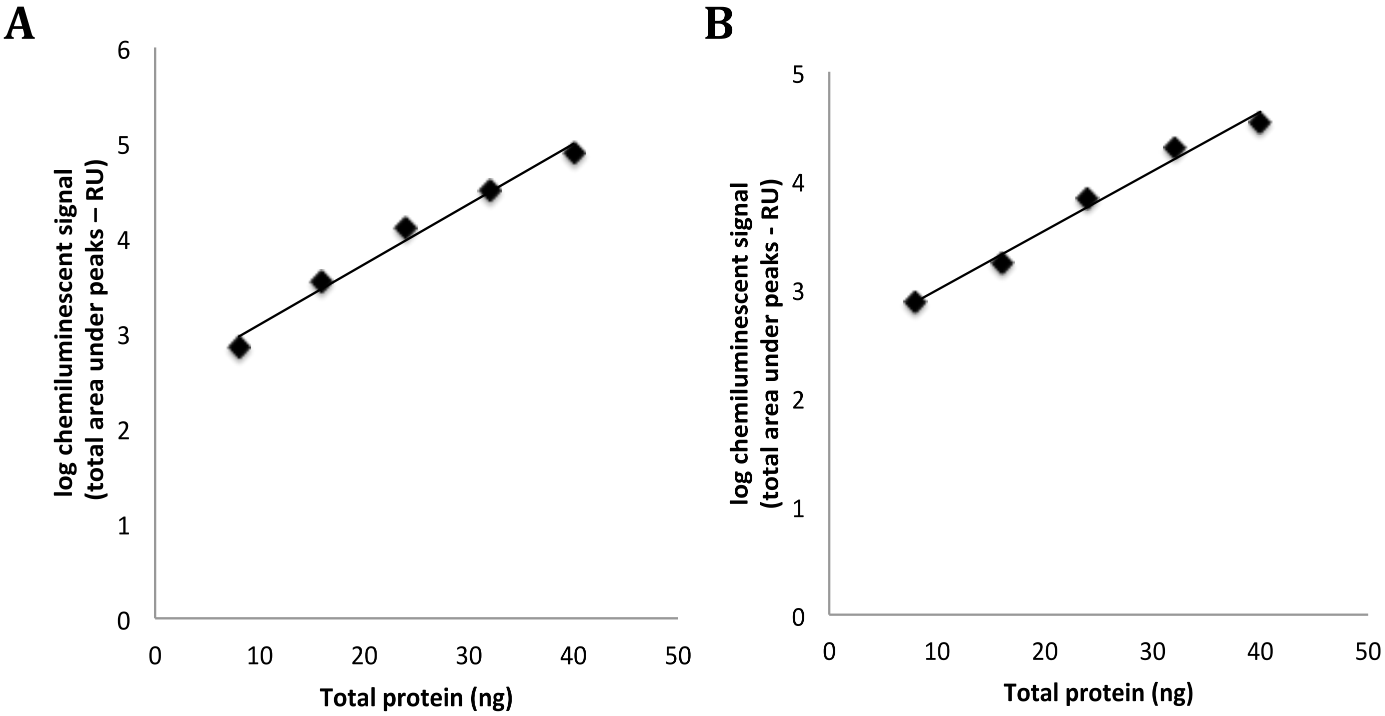

Supplementary Figure 2. Log chemiluminescent signal calculated from area under all peaks for total **(A)** ERK and **(B)** AKT plotted against total protein from pooled lung and tumour samples. R^2 = 0.98 for both trendlines. Limit of detection for both assays was 8ng total protein.
